# Design of Functionally Graded Alloys for Locks Highly Resistant to Ultrasonic Detector Attacks

**DOI:** 10.3390/ma19112268

**Published:** 2026-05-27

**Authors:** Luka Matić, Antonio Petošić, Viktor Šunde, Željko Ban

**Affiliations:** 1 Research Department, Končar Electrical Engineering Institute, 10000 Zagreb, Croatia; 2 Department of Control and Computer Engineering, Faculty of Electrical Engineering and Computing, 10000 Zagreb, Croatia; zeljko.ban@fer.hr; 3 Department of Electroacoustics, Faculty of Electrical Engineering and Computing, 10000 Zagreb, Croatia; antonio.petosic@fer.hr; 4 Department of Electric Machines, Drives and Automation, Faculty of Electrical Engineering and Computing, 10000 Zagreb, Croatia; viktor.sunde@fer.hr

**Keywords:** functionally graded materials, 3D printing, ultrasound lock decoding, high-security pin-tumbler locks, mathematical modeling, *simplex* optimization

## Abstract

Mechanical locks have not been fully replaced by electrical locks and are still being researched and improved, along with advanced electronic methods of attack. Moreover, reading pin lengths by detecting their natural frequencies (lock decoding) to forge copies of a legitimate key can be done quickly using active or passive ultrasonic detectors. One possible method of defence against them is manufacturing lock pins using functionally graded materials (FGMs). A pin’s natural frequency (in the range 100 kHz–1 MHz) and hence its ultrasonic pulse transit/reflection time can be correlated to its length if it is made of a homogeneous material. The idea is to design pins made of functionally graded alloys to achieve equal natural frequencies, but also desired positions of standing wave nodes regardless of pin length. To calculate the composition of the FGM alloy, we must first develop mathematical models of a pin’s vibrations. Two simple and fast mathematical models are first derived from the finite-element model (FEM) of a pin. These models are used in an optimization procedure based on the Nelder–Mead *simplex* method to calculate optimal profiles of Young’s modulus and density along a pin’s longitudinal axis. A successful optimization procedure for 10 key pin lengths is performed to make a pin-tumbler lock resistant to ultrasonic attacks.

## 1. Introduction

Mechanical locks have been continuously improved [1,2,3], while the mechanical assemblies of electric locks still present unresolved challenges. For this reason, mechanical locks are still an important link in the chain of security. The evolution of mechanical lock design has been accompanied by a parallel advancement in defeat and exploitation techniques [4,5,6,7,8]. Methods [7,8] use audible sound, recorded at a distance, and mention possible defences (playing random noises) only briefly. As an emerging field, ultrasonic methods offer vast potential for growth and innovation across both attacks and defences.

To improve physical security, this paper focuses on safeguarding mechanical locks from active and passive ultrasonic exploitation. No major changes will be made to the mechanical assembly. The shapes and sizes of all other parts, including the driver pins, will stay the same. Only the material composition of the key pins will be altered. This way, a high-security lock’s protection against standard mechanical attacks [9,10,11] (e.g., bumping, picking or raking using Kopsch’s tools, mechanical decoding with “Sputnik” tool, drilling, etc.) in the form of pick-proof pins, trapping calottes (*Fangkalotten*) [2,3], double pins, pins in multiple planes, cascaded locks (*Kaskadensystem*) [3,9], anti-drilling rods, etc., will be kept, and extra protection will be added.

**Figure 1:**
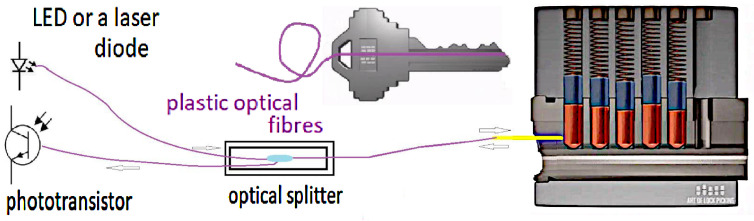
A pin-tumbler lock and a passive ultrasonic detector.

Active ultrasonic detectors [12] determine lock pin lengths by measuring the time delay of the ultrasound echo bouncing off the tip of each pin. Passive ultrasonic detectors [13], which are cheaper, register vibrations on a pin’s lateral surface (Figure 1) using a laser beam perpendicular to the pin. The laser microphone records pin vibrations at the pin’s impact on the plug/rotor, after the pin’s spring is compressed and abruptly released. The measured natural frequencies are then correlated with the lengths of the pins.

For both types of ultrasonic attacks to succeed, Young’s modulus *E* and density ρ of key pins must be known to the attacker, and pins of different lengths must have different natural frequencies. Both key pins and driver pins on all known high-security pin-tumbler locks are always made of homogeneous metal alloys, most often brass or steel. Driver pins are all the same length, while key pins from one manufacturer are usually made in 10 standard lengths. Every key has a code, like 2-4-6-5-8 for a five-pin lock that determines the length of its pins. Since the natural vibration frequency of a pin changes based on how long it is, attackers can use ultrasonic waves to easily identify the pins and exploit the lock.

A lock resistant to ultrasonic attacks should have key pins with equal natural frequencies regardless of their lengths. Furthermore, pointing the laser probe at a standing wave node on the pin will prevent the passive ultrasonic attack from succeeding. This is why the key pins should be made of alloys with variable E(x) and density ρ(x) along the pin’s longitudinal axis *x*.

**Figure 2:**
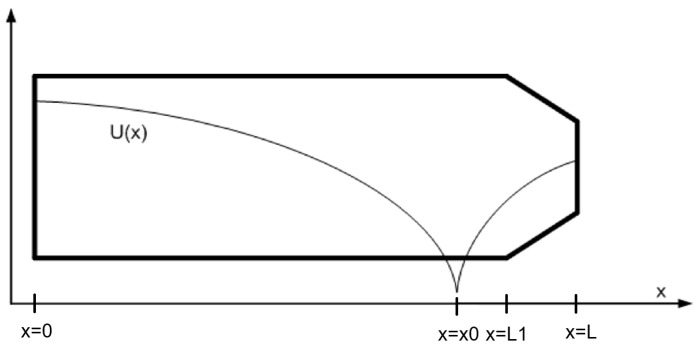
Standing wave and simplified shape of a key pin.

The natural frequency *f* of longitudinal vibrations of a thin rod [14] of length *L* made of a homogeneous material is

(1) $$f=\frac{1}{2L}\sqrt{\frac{E}{\rho },}$$

where *E* is Young’s modulus and ρ is density. While using different homogeneous alloys allows all 10 pins of varying lengths to achieve an identical frequency, this approach alters the positions $x_{0}$ (Figure 2) of the standing wave nodes. A node is then fixed to a point $x_{0}<L/2$ and cannot be moved. If it is possible to move $x_{0}$ to a point close to a pin’s tapered tip, like in Figure 2, which is a spot where a laser probe will be pointing, it will be more difficult for an attacker to record a useful signal because of the small amplitude of vibrations.

Even for a homogeneous material, Equation (1) is valid only if the rod can be considered “thin” (i.e., if the ratio L/d>1.7 for error < 0.1%), and if cross-section A(x) is constant. Gradient dA(x)/dx increases the speed of sound v(x), thus increasing the frequency *f* and moving the node point $x_{0}$ to the left from L/2. Its preferred position is actually to the right, as shown in Figure 2.

Instead of traditional homogeneous alloys, this paper suggests using Functionally Graded Materials (FGMs) to manufacture key pins, building on research conducted since the mid-1980s [15]. Many new FGMs along with new methods of manufacturing have been invented since then [16,17,18,19]. Originally deployed in the construction of space shuttle wings, this approach aimed to synthesize the tensile strength of steel with the superior thermal properties of ceramic materials. Because conventional lamination yields an unacceptably weak structure, a functionally graded transition, progressing from pure ceramic at the surface to bulk metal within, was engineered to maximize mechanical and thermal performance.

FGM will improve a lock’s resistance to ultrasonic attacks, because by varying E(x) and ρ(x) along the x-axis, both a pin’s natural frequency and position of standing wave node can be manipulated. This way, it is difficult to correlate a pin’s natural frequency to its length. The method to produce such FGM pins, i.e., 3D printing from metal powders, is already well established, the so-called directed energy deposition (DED) method that uses several different metal powders, mixed at different ratios on every point along the x-axis, to achieve the desired E(x) and ρ(x) values. The volume ratios of metal powders are calculated from E(x) and ρ(x) according to Voigt’s model.

Mechanical vibrations on FGM structures [20,21,22] have been modeled by different application-specific models, which is a further contribution of this paper, along with a method to optimize E(x) and ρ(x) profiles. This means that mathematical models of pins’ vibrations on a mechanical transmission line with variable E(x), ρ(x) and A(x) have to be devised first; this is done in Section 2. Simplified models, appropriate for use in an optimization procedure (i.e., fast enough to calculate 500–1000 successive simulations in a reasonable time), have been derived in Section 3. Initial calculations for pins with two distinct sections (E(x) and ρ(x) as step functions) as a starting point for the optimization procedure (to reach set referent values both for *f* and $x_{0}$) are done in Section 4, along with the transition to gradual E(x) and ρ(x) derived from Butterworth polynomials. An optimization procedure to calculate the optimal E(x) and ρ(x) profiles is defined and performed in Section 5. Final adjustments to ameliorate the resistance against ultrasonic attacks (by compensating for short stick effects and introducing some random variables) are performed in Section 6. Preliminary experimental results on the equipment and samples currently available and a discussion of necessary improvements are presented in Section 7.

The main novel elements of this paper are:

- Use of FGM alloys for manufacturing key pins to improve pin-tumbler locks’ resistance to ultrasonic attacks.
- New fast mathematical models for one-dimensional axial vibrations on mechanical lines.
- Optimization procedure to calculate FGM pins, with dimensions pre-defined by an existing lock assembly.

## 2. Mathematical Models of Pins Vibrations

Key pins excited by an axial impulse (from a piezo transducer probe of an active ultrasonic decoder, or an impact with a plug/rotor in front of a laser probe of a passive ultrasonic decoder) behave as a mechanical transmission line with free ends. Both ends of the key pins are not affixed, and the loading spring is very soft compared to a metal pin; hence, the termination impedance can be considered practically infinite at both ends. According to [23], the error in natural frequency of a one-dimensional thin-rod axial model is less than −2.7% for the smallest key pins (i.e., for pins with ratio L/d=1.4, which is the lowest value among all standard pin-tumbler locks). The difference in natural frequencies between the two shortest standard pin lengths (pin-1 and pin-2, pin numbering goes up to pin-10 for the longest pin, for a lock with 10 pin lengths) is usually higher than 10%.

**Figure 3:**
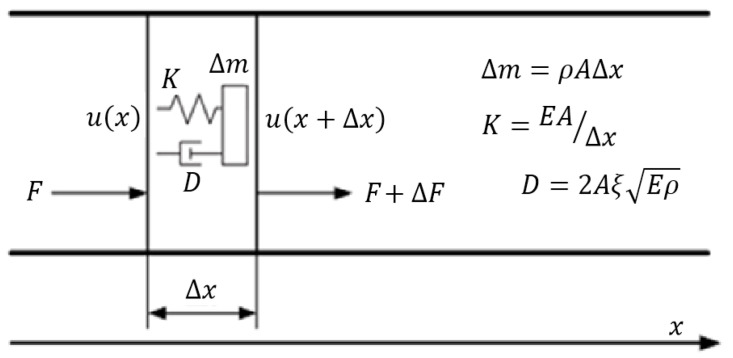
A mechanical transmission line.

The partial differential equation (PDE) for a one-dimensional mechanical line (shown in Figure 3. *K* is stiffness in [N/m] of a line segment of width Δx, *A* is cross-sectional area, *F* is force on segment Δx, Δm is its mass, *D* is a damping coefficient in [Ns/m], and ξ is a dimensionless damping factor) [21,24] with variable parameters (2) cannot be solved analytically in the general case.

(2) $$\frac{\partial }{\partial x}\left(E\left(x\right)A\left(x\right)\frac{\partial u\left(x,t\right)}{\partial x}\right)=2\xi_{l}A\left(x\right)\sqrt{E(x)\rho (x)}\frac{\partial u\left(x,t\right)}{\partial t}+\rho \left(x\right)A\left(x\right)\frac{\partial^{2}u\left(x,t\right)}{\partial t^{2}}$$

Damping factor ξ for metallic alloys is very low (in the order of $10^{-3}$), hence its term (containing $\xi_{l}$, a damping factor per unit length) in (2) is neglected. It does not influence the natural frequency and position of standing wave nodes. Equation (2) is then written in a more appropriate form:

$$\frac{\partial E_{A}\left(x\right)}{\;\;\partial x}\frac{\partial u(x,t)}{\partial x}+E_{A}\left(x\right)\frac{\partial^{2}u\left(x,t\right)}{\partial x^{2}}=\rho_{A}\left(x\right)\frac{\partial^{2}u\left(x,t\right)}{\partial t^{2}}$$

(3) $$E_{A}\left(x\right)=E\left(x\right)A\left(x\right),\;\;\;\;\rho_{A}\left(x\right)=\rho \left(x\right)A\left(x\right)$$

### 2.1. FEM

FEMs are accurate but slow. An FEM is coded in MATLAB R2020b to be used as a reference for the evaluation of faster and simplified models designed in Section 3. Sampling time is $\Delta t=T_{d}$, number of finite elements is N=500, pin length is *L*, and length of one FEM segment is Δx=L/N=l. Signal u(x,t) discretized in space and time is u(j,k). Using standard Euler’s approximations for derivatives, the following Equations (4)–(6) derived from (3) will define the FEM.

$$u\left(j,k+1\right)=w^{2}v^{2}\left(j\right)\cdot u\left(j+1,k\right)+\left[w^{2}v^{2}\left(j\right)\cdot \left(a\left(j\right)-2\right)+2\right]\cdot u\left(j,k\right)+$$

(4) $$+w^{2}v^{2}\left(j\right)\cdot \left(1-a\left(j\right)\right)\cdot u\left(j-1,k\right)-u\left(j,k-1\right)$$

(5) $$w=\frac{T_{d}}{l},\;\;a\left(j\right)=1-\frac{E\left(j-1\right)A\left(j-1\right)}{E\left(j\right)A\left(j\right)}$$

(6) $$v\left(j\right)=\sqrt{\frac{E(j)}{\rho (j)}},\;\;x\left(j\right)=\frac{jL}{N}=jl$$

The boundary condition for the left-hand (j=1) free end excited by impact force $F_{1}$ is described by (7), and analogously for the right-hand free end (j=N).

(7) $$u\left(1,k+1\right)=\left(F_{1}\left(k\right)+\left(u\left(2,k\right)-u\left(1,k\right)\right)\frac{E_{1}A_{1}}{l}\right)\frac{T_{d}^{2}}{\rho_{1}A_{1}l}+2u\left(1,k\right)-u\left(1,k-1\right)$$

A condition (8) for a minimum sampling time $T_{d}$ is required for a stable FEM simulation.

(8) $$T_{d}<\begin{array}{c} \min\\ j \end{array}\sqrt{\frac{\rho (j)}{E(j)}}\;l$$

### 2.2. Some Exact Solutions of the PDE

Variables *x* and *t* can always be separated, because standing waves will be formed on a line with two free ends. Furthermore, amplitude U(x) is important for optimization, not the instantaneous value of the longitudinal vibrations u(x,t)=U(x)exp(iωt). Boundary conditions will thus always be dU(0)/dx=0 and dU(L)/dx=0. For the simplest case, i.e., a thin pin with constant *E*, ρ, and *A*, the natural frequency is (1).

#### 2.2.1. Constant $E_{0}$ and $\rho_{0}$, Exponential A(x)

For a pin with a cross-section described with an exponential function $A\left(x\right)=A_{0}exp\left(-hx\right)$, and constant *E* and ρ, the exact natural frequency can be calculated by the analytical solution of (3):

(9) $$f=\frac{1}{2L}\sqrt{\frac{E_{0}}{\rho_{0}}}\sqrt{1+\frac{h^{2}L^{2}}{4\pi^{2}}}$$

This natural frequency is always higher than for the constant cross-section (1). This indicates that the gradient dA(x)/dx increases the propagation speed v(x) in the same material. For a cylindrical driver pin, *L* = 6.10 mm, *d* = 2.92 mm, with constant $E_{0}$ = 120 GPa and $\rho_{0}$ = 8000 kg/$m^{3}$, the calculated (1) natural frequency is *f* = 317.5 kHz. The FEM simulation result is *f* = 317.0 kHz. For a tapered pin of the same dimensions, with *h* = 400 $m^{-1}$ (right-hand end diameter reduced to d(L) = 0.86 mm), the calculated (9) natural frequency is *f* = 340.6 kHz. The FEM simulation gives *f* = 341.0 kHz.

#### 2.2.2. Constant $A_{0}$, Exponential E(x) and $\rho_{(}x)$

For the Young modulus and density described as $E\left(x\right)=E_{0}exp\left(ax\right)$ and $\rho \left(x\right)=\rho_{0}exp\left(cx\right)$, propagation speed and natural frequency can also be calculated by the analytical solution of Equation (3):

(10) $$v\left(x\right)=\sqrt{\frac{E_{0}}{\rho_{0}}}\cdot exp\left(\frac{a-c}{2}x\right)$$

(11) $$f=\frac{c-a}{4}\sqrt{\frac{E_{0}}{\rho_{0}}}\cdot \frac{1}{exp\left(\frac{c-a}{2}L\right)-1}$$

For a driver pin as in Section 2.2.1, with *a* = 200 $m^{-1}$ and *c* = −100 $m^{-1}$ (right-hand parameters E(L) = 406 GPa, ρ(L) = 4347 kg/$m^{3}$), the calculated (11) natural frequency is *f* = 484.6 kHz. The FEM simulation result is *f* = 483.5 kHz. The results confirm sufficient precision of FEMs with *N* = 500.

These equations can also be analytically calculated by integration of time $\frac{T}{2}=\int_{0}^{L}\frac{dx}{v(x)}$, which will be the basis for devising fast and simple models of FGM key pin vibrations in the next section.

## 3. Fast Mathematical Models for Optimization of FGM Key Pin Profiles

Starting from PDE (3) and the standing wave equation u(x,t)=U(x)exp(iωt), the ordinary differential equation (ODE) (12) is derived:

(12) $$\frac{d^{2}U\left(x\right)}{{dx}^{2}}=-\frac{dE_{A}\left(x\right)}{dx}\frac{1}{E_{A}\left(x\right)}\frac{dU\left(x\right)}{dx}-\omega^{2}\frac{\rho_{A}\left(x\right)}{E_{A}\left(x\right)}U\left(x\right)$$

**Figure 4:**
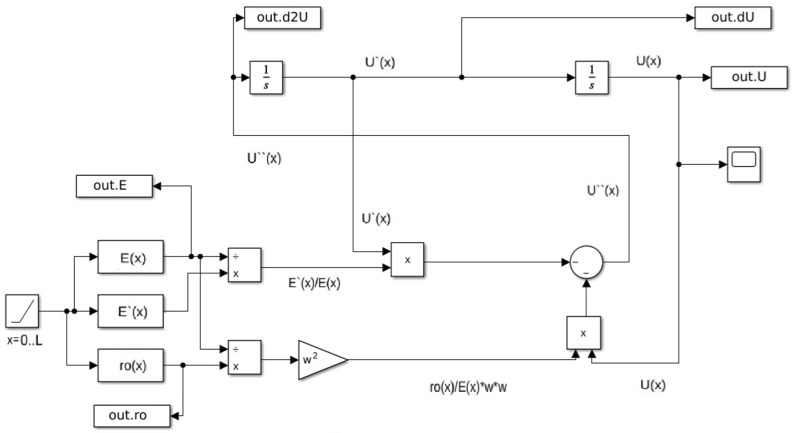
ODE model for *Simulink*.

**Figure 5:**
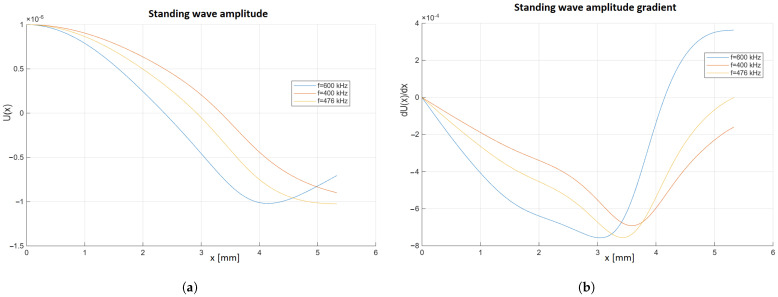
Results of ODE model simulation: (**a**) standing wave amplitudes for three different frequencies; (**b**) standing wave amplitude gradients for three different frequencies.

This is not analytically solvable for every $E_{A}\left(x\right)$ and $\rho_{A}\left(x\right)$ function. However, for a known $E_{A}\left(x\right)$ and $\rho_{A}\left(x\right)$, the solution (*f* and $x_{0}$) can be found with sufficient precision with less than 10 successive simulations, using a *Simulink* model in Figure 4. Variable *x* is treated as time. Integrators work with fourth-order Runge–Kutta with a constant time step (actually the FEM element length l=L/N). The initial value on integrator U(0) is set to the max value (1 μm in this case), and on the other integrator to $U^{\prime }\left(0\right)=0$ (this is one boundary condition). Hence, if ω=2πf is set to the pin’s correct natural frequency, the second boundary condition $U^{\prime }\left(L\right)=0$ will be met at the end of the simulation (Figure 5b) for f = 476 kHz. The standing wave node position $x_{0}=2.92$ mm is then read directly from U(x) in Figure 5a.

One *Simulink* simulation takes 0.3 s, meaning 3 s will be needed to establish the $x_{0}$, *f* and U(x) profile. This is 10 times faster than a single FEM simulation in Matlab (cca. 30 s). This model is more useful for checking *f* and $x_{0}$ calculated using another method, because it is then 100 times faster than the FEM, and its precision (i.e., *f*, $x_{0}$ and U(x)) is the same as the FEMs. However, a faster model is required for FGM pins’ profile optimization procedure, since 500–1000 successive simulations are needed in *simplex* optimization of one pin to reach an optimum profile.

### 3.1. Speed Profile v(x) Approximation

If $A\left(x\right)=A_{0}$ is constant, the ultrasound speed at point *x* can be calculated as $v\left(x\right)=\sqrt{E(x)/\rho (x)}$. Natural frequency f=1/T, and standing wave node position $x_{0}$, similarly to (11), can then be calculated by numerical integration of time (13):

(13) $$\frac{T}{2}=\int_{0}^{L}\frac{dx}{v(x)},\frac{\;T}{\;4}=\int_{0}^{x_{0}}\frac{dx}{v(x)}=\int_{x_{0}}^{L}\frac{dx}{v\left(x\right)}$$

The influence of dA(x)/dx along with E(x) and ρ(x) on speed v(x) is difficult to calculate analytically. The main idea is to approximate the increase in speed $v\left(x\right)=\sqrt{E(x)/\rho (x)}$ at the pin’s tip (where dA(x)/dx≠0) accurately enough; then, (13) can be used as a fast model for the optimization procedure. Similar approximations were used in [25] to solve PDEs of homogeneous sticks with variable cross-sections with sufficient precision. This way, *f* and $x_{0}$ can be quickly calculated using (13), and then model (12) can be used to calculate U(x) and to confirm the values of *f* and $x_{0}$. The simplest approach is to use an exponential function (14) where $v_{a}\left(x\right)=\sqrt{E(x)/\rho (x)}$ as a speed correction factor at the tip ($x>L_{1}$).

(14) $$v\left(x\right)=v_{a}\left(x\right)exp\left(b\left(x-L_{1}\right)\right)$$

Even if v(x) does not match exactly at every point on the x-axis, but *f* and $x_{0}$ match the ODE model (12), this will be precise enough for the pin optimization procedure. For a pin of certain dimensions (length *L*, and a tip of constant dimensions for all 10 standard lengths of a particular lock manufacturer), it will be shown that coefficient b=b(L) can be calculated to achieve sufficient precision. Standard key pins of the locksmithing company *Schlage* will be further used as a case study.

### 3.2. Calculation of b Coefficient

**Figure 6:**
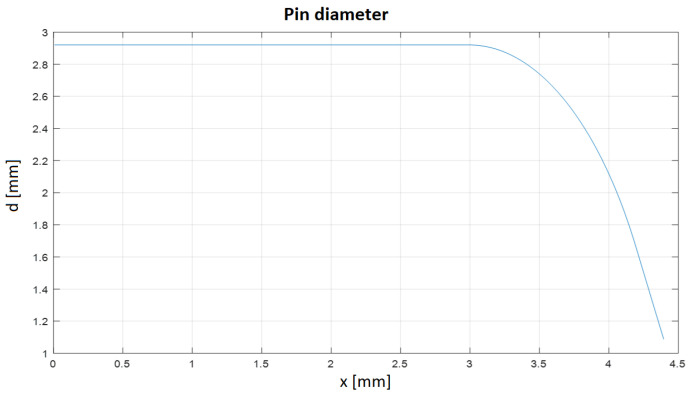
A key pin’s diameter profile.

Two straight lines and one circular arc approximate the tip of a standard *Schlage* key pin, as shown in Figure 6. The coefficient *b* was calculated for 10 homogeneous pins (made of brass, $v\left(x\right)=v_{0}=3870$ m/s). It will then be shown that this exponential correction of speed is also accurate for arbitrary variable E(x) and ρ(x). Pulse propagation time from x=0 to x=L is

$$t\left(L\right)=\int_{0}^{L}\frac{dx}{v(x)}=\int_{0}^{L_{1}}\frac{dx}{v_{0}}+\int_{L_{1}}^{L}\frac{dx}{v_{0}\;exp\left(b\left(x-L_{1}\right)\right)}=$$

(15) $$=\frac{L_{1}}{v_{0}}+\frac{1}{bv_{0}}\left(1-exp(b\left(L-L_{1}\right))\right)=T/2,$$

where natural frequency is f=1/T, propagation time from one end to standing wave node is $t\left(x_{0}\right)=t\left(L\right)/2$, and $x_{0}=v_{0}\cdot t\left(x_{0}\right)$ for $x_{0}<L_{1}$.

The following substitutions will be introduced (16). The length of a tip $d_{sch}=1.4$ mm is constant for all 10 pins; $L_{C}$ is dependent on *L*.

(16) $${\;\;d}_{sch}=L-\;L_{1},\;{\;\;L}_{C}=\frac{1}{b}\left(1-exp(b\left(L-L_{1}\right))\right)$$

From (15) and (16), the implicit Equation (18) is derived.

(17) $$\;\;\;f=\frac{v_{0}}{2}\cdot \frac{1}{L_{1}+L_{C}}\;,\;\;x_{0}=\frac{1}{2}\left(L_{1}+L_{C}\right)\;$$

(18) $$\;\;b=\frac{1}{v_{0}/\left(2f\right)-L_{1}}\left(1-exp(bd_{sch})\right)$$

Equation (18) will be used to iteratively calculate the value of *b* according to the following procedure:

(1) For a standard pin length *L*, and $A\left(x\right)=d^{2}\left(x\right)\pi /4$ with d(x) according to Figure 6, run a *Simulink* simulation according to (12) and Figure 4. This yields *f* and $x_{0}$.
(2) Using *f*, iteratively calculate b=b(L) from (18).
(3) Calculate $L_{C}=L_{C}\left(b\right)$ from (16).
(4) Take the next standard value for *L* and go to step 1.

This way, for 10 standard *Schlage* pin lengths,

 *L* [mm] = [4.19 4.57 4.95 5.33 5.72 6.10 6.48 6.86 7.24 7.62]

the following values of *b* and $L_{C}$ are obtained:

 *b* [$m^{-1}$] = [516 520 524 528 532 537 541 545 549 553]

 $L_{C}$ [mm] = [1.00 1.00 0.99 0.99 0.98 0.98 0.98 0.97 0.97 0.97]

### 3.3. Comparison of Two ODE Models

An approximation of the initial ODE model (12) with variable A(x), E(x) and ρ(x) (called “A” model) is compared with the ODE model with constant cross-section $A\left(x\right)=A_{0}$, and variable E(x) and ρ(x) (called “v” model). In the second model, the E(x) will be multiplied by the factor $exp(b\left(x-L_{1}\right))$ for $x>L_{1}$, and ρ(x) will be divided by the same factor, which will effectively multiply speed $v\left(x\right)=\sqrt{E(x)/\rho (x)}$ by that factor along the pin’s tip.

#### 3.3.1. Natural Frequencies of Brass *Schlage* Pins

**Figure 7:**
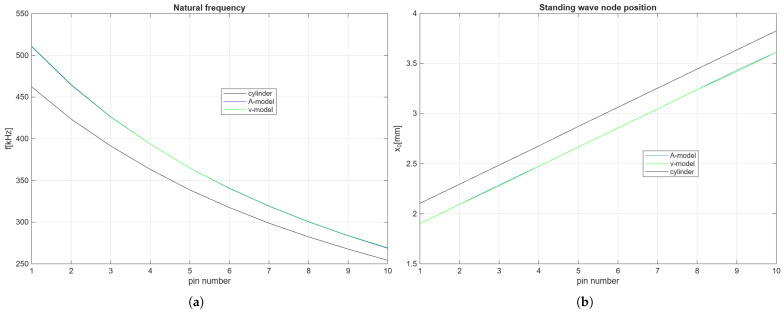
Results of simulation of 3 different ODE models for 10 pin lengths: (**a**) natural frequencies of pins, (**b**) positions of standing wave nodes on pins.

Pins are homogeneous and made of brass ($E_{0}$ = 120 GPa, $\rho_{0}$ = 8000 kg/$m^{3}$). The results of simulations of ODE models “A” and “v” in Figure 7 show that there are very small differences between models (maximum frequency error is 0.88 kHz, and maximum standing wave node position error is 0.015 mm) for homogeneous pins. For comparison, ODE model simulations of cylindrical pins (such as driver pins, without tapered tips) are also performed, showing the frequency error increases up to 10% (for pin-1) if the tapered tips are neglected.

#### 3.3.2. Comparison of “A” and “v” Models of FGM Pins

**Figure 8:**
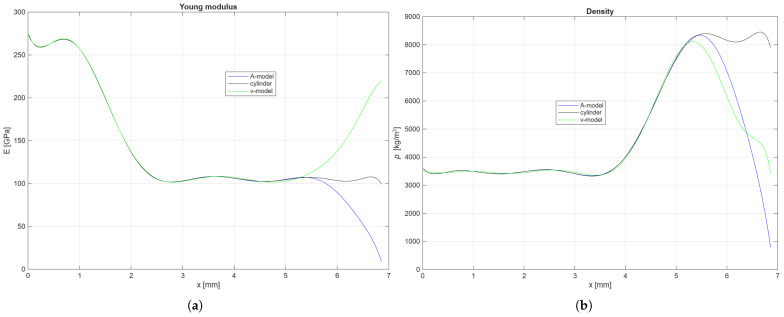
Variable profiles of an FGM pin. (**a**) Young’s modulus, (**b**) density.

ODE models simulations are performed for an FGM pin of length L= 6.86 mm, as in the previous subsection. The natural frequency of the pin is f= 364 kHz, the node position is $x_{0}$ = 3.98 mm, and the accuracy remains the same. Variable E(x) and ρ(x) in Figure 8 are shown multiplied by factor $A\left(x\right)/A_{0}$ for the “A” model, and with the exponential factor for the “v” model. Both ODE models also show equally high accuracy compared with the FEM.

On the other hand, as shown in Figure 8a, the ratio of the maximum and minimum value of $E_{A}\left(x\right)$ of the “A” model can be much higher than the ratio of respective values of the “v” model. This can increase numeric errors at ODE model integrators in the “A” model. This is why, for certain E(x) profiles, the accuracy of the “A” model decreases compared to FEM and “v” models.

### 3.4. Testing of Fast Time Integrator Model

Since the accuracy of the ODE “v” model is confirmed, and hence the accuracy of speed correction with the exponential factor, fast time integrator model (13) can now be tested for accuracy, since it uses that same approximation. For a pin with $E_{v}\left(x\right)$ and $\rho_{v}\left(x\right)$ profiles as in Figure 8, the fast time integrator model results are *f* = 403 kHz and $x_{0}$ = 4.07 mm. Therefore, the frequency error is >10%.

**Figure 9:**
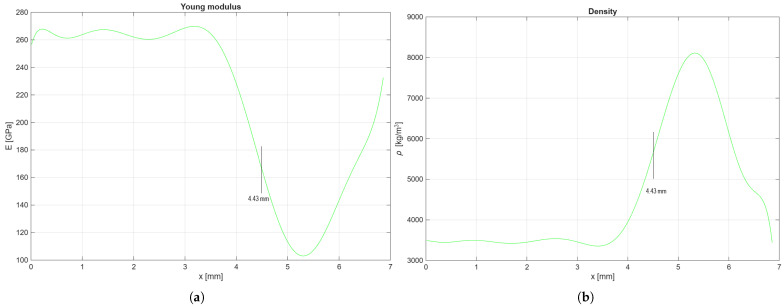
Profiles of an FGM pin with a transition point at the standing wave node. (**a**) Young’s modulus, (**b**) density. Both profiles are “v” variant, with exponential correction of speed at the pin tip.

On the other hand, for a pin with $E_{v}\left(x\right)$ and $\rho_{v}\left(x\right)$ profiles as in Figure 9 (transition points of both *E* and ρ are now close to $x_{0}$), the accuracy is much better. ODE “v” model gives *f* = 476.7 kHz, $x_{0}$ = 4.43 mm, while the fast time integrator gives *f* = 476.2 kHz, $x_{0}$ = 4.43 mm.

The accuracy of this model increases as transition points approach the standing wave node. It is shown in Section 5 that it is possible to set the optimization objective and penalty functions so that the node position $x_{0}$ falls close to both transition points at the end of the optimization procedure.

This model is still accurate enough for the optimization procedure and is the most appropriate for it. It can complete 1000 simulations in MATLAB in 3 s. When the optimum point is reached, the final result can be confirmed by one simulation using the ODE “v” model.

## 4. Initial Calculations for FGM Pins

There is more than one way to achieve the optimization objective, but the *simplex* method’s convergence is not always guaranteed. This is why its initial vectors must be properly set. The following procedure to achieve reliable convergence is now described.

### 4.1. Setting the Initial Parameters for Optimization

Setting the initial parameters for optimization is performed according to the following algorithm:

(1) A desired distance of the standing wave node from the pin cone $d_{0}=L_{1}-x_{0}$ is first set. Let $d_{0}=$ 1 mm.
(2) Calculate (according to (16)–(18)), for a homogeneous pin (e.g., made of brass, like in Section 3.2), length $L_{ref}=L_{C}+2d_{0}+d_{sch}$ = 4.395 mm (since $L_{C}$ depends on *L*, a few iterations may be needed).
(3) For this $L_{ref}$, also according to (18), calculate $f=v_{2}/2/\left(L_{ref}-d_{sch}+L_{C}\right)$ = 476 kHz, for $v_{2}$ defined by initial homogeneuous material (brass), i.e., 3800 m/s.

**Figure 10:**
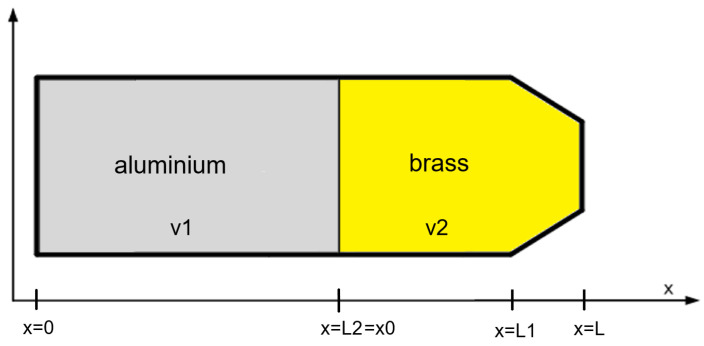
An example of a two-section key pin.

This is not the length of a standard *Schlage* pin (it falls between pin-1 and pin-2). It will be used to set a reference for natural frequency (476 kHz) and node position ($d_{0}$ = 1 mm left from the tapered tip). The idea is to keep the right-hand side the same (made of brass), and change the composition of the homogeneous alloy on the left-hand side (e.g., like Figure 10), so its speed of sound $v_{1}$ satisfies the equation

(19) $$\frac{v_{1}}{L-d_{0}-d_{sch}}=\frac{v_{2}}{L_{ref}-d_{0}-d_{sch}}$$

(4) This way, the wave propagation time on the left-hand side remains the same, keeping the frequency *f* and node position $d_{0}=L_{1}-x_{0}$ unchanged for every standard pin of length *L*:
  *L* [mm] = [4.19 4.57 4.95 5.33 5.72 6.10 6.48 6.86 7.24 7.62]
  $v_{1}$ [km/s] = [3.41 4.13 4.86 5.58 6.32 7.05 7.77 8.50 9.22 9.95]

FEM simulations confirm this approach; natural frequency and node positions remain the same for all 10 standard lengths.

### 4.2. Using Butterworth Polynomials to Improve the Structure

A structure like in Figure 10, with an abrupt transition, may be difficult to implement in practice. The reason is a problem with achieving sufficient adhesion along the separation plane ($x=L_{2}$), along with a possible problem of fitting their different crystal grids. Furthermore, when attacked using an active ultrasonic detector (ultrasonic pulse injected at the pin’s tip x=L), there will be a reflection from the separation plane ($x=L_{2}$), and then another from the flat, left-hand end (x=0). Their delay times will be equal for any pin length, but the ratio of amplitudes and phases of these two reflected pulses will be different for different materials on the left-hand side. This is another reason why the transition must be made gradually (a gradual transition will not create reflections), so it can be realized using methods including 3D printing/sintering from metal powders.

**Figure 11:**
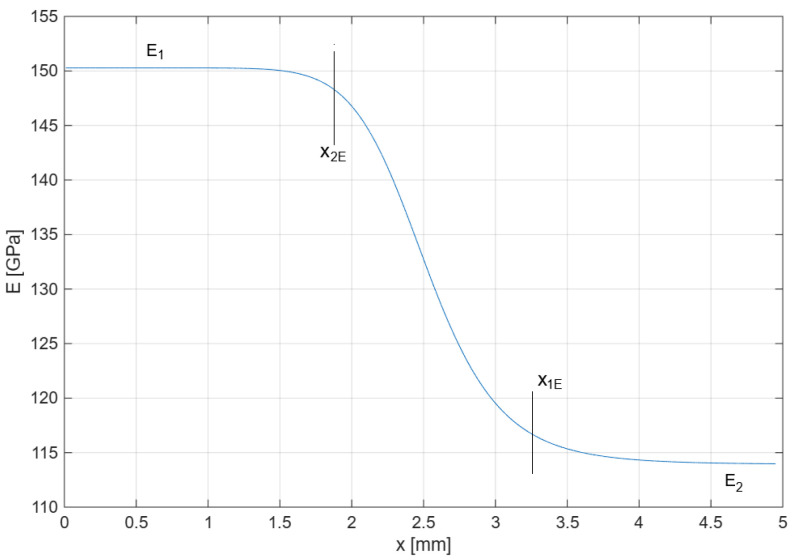
A gradual transition of Young’s modulus.

Butterworth polynomial magnitudes (20) will be used to implement gradual transitions of E(x) (in Figure 11), and analogously, ρ(x) as well. The transitions are thus located in the bulk, not close to surface like in usual planar FGM structures described in [15].

(20) $$E\left(x\right)=E_{1}\frac{1+{\left(\frac{x}{x_{1E}}\right)}^{2n_{E}}}{1+{\left(\frac{x}{x_{2E}}\right)}^{2n_{E}}}\;\;,\;\;\rho \left(x\right)=\rho_{1}\frac{1+{\left(\frac{x}{x_{1\rho }}\right)}^{2n_{\rho }}}{1+{\left(\frac{x}{x_{2\rho }}\right)}^{2n_{\rho }}}$$

(21) $$\;\;\;\;{\;\;E}_{2}=E_{1}{\left(\frac{x_{2E}}{x_{1E}}\right)}^{2n_{E}}\;,\;{\;\rho }_{2}=\rho_{1}{\left(\frac{x_{2\rho }}{x_{1\rho }}\right)}^{2n_{\rho }}$$

Equation (20) shows the magnitudes of Butterworth filter transfer functions, with ±3 dB points at $x_{1}$ and $x_{2}$. Their exponents $n_{E}$ and $n_{\rho }$, however, don’t have to be integers. To estimate the width of the transition region $\Delta x_{trans}$ for profile E(x) (to set maximum acceptable values of exponent $n_{E}$), a tangent in the inflection point $x_{inf}$ is used, in a span between $E_{1}$ and $E_{2}$ (22), and analogously for profile ρ(x).

(22) $$E^{\prime }\left(x_{inf}\right)=\frac{E_{1}}{x_{inf}}\left({\left(\frac{x_{2E}}{x_{1E}}\right)}^{2n_{E}}-1\right)\frac{4n_{E}^{2}-1}{8n_{E}}$$

(23) $$x_{inf}=x_{2E}{\left(\frac{2n_{E}-1}{2n_{E}+1}\right)}^{\frac{1}{2n_{E}}},\;\;\Delta x_{trans}=\frac{E_{2}-E_{1}}{E^{\prime }\left(x_{inf}\right)}$$

From (22) and (23) it can be estimated that $\Delta x_{trans}$ will be $\Delta x_{trans}$ >0.4 mm if $n_{E},n_{\rho }<20$, hence it will be a bound for one of the penalty functions for *simplex* optimization. Standard metal powders with a particle diameter of 10 μm are easily available to achieve a sufficiently gradual transition. Furthermore, the lower bound will be $n_{E},n_{\rho }>2$, because if the exponent is too low, then Equation (21) for $E_{2}$ and $\rho_{2}$ is not valid.

### 4.3. Setting the Initial Vector for Simplex Optimization

The optimization vector *h* will be defined as follows:

(24) $$h=[\;\Delta x_{E}\;\;\Delta x_{\rho }\;\;x_{mid}\;\;\rho_{1}\;\;\rho_{2}\;\;E_{1}\;\;E_{2}\;]$$

$$x_{2E}=x_{mid}-\Delta x_{E}\;,\;x_{1E}=x_{mid}+\Delta x_{E}$$

$$x_{2\rho }=x_{mid}-\Delta x_{\rho }\;\;,\;x_{1\rho }=x_{mid}+\Delta x_{\rho }$$

The initial values of the *h* vector, for every pin length *L* and corresponding speed $v_{1}$ calculated by (19) are (25). Value $g_{inf}=\left|E^{\prime }\left(x_{inf}\right)\right|/E_{1}$ is an acceptable gradient at the inflection point.

$$\begin{array}{c} v_{2}=3800\;m/s,\;\rho_{2}=8000\;\mathrm{kg}/m^{3},\;E_{2}=\rho_{2}v_{2}^{2},\;n=10\\ \rho_{1}=\rho_{2}v_{2}/v_{1},\;\;E_{1}=E_{2}v_{1}/v_{2},\;\;L_{2}=L-d_{0}-d_{sch},\;\;g_{inf}=1500\;m^{-1}\;\; \end{array}$$

(25) $$\Delta x_{E}=\left|L_{2}\left(1-k\right)\right|,\;k=exp\left(\frac{ln(1+2g_{inf}L_{2}/n)}{4n}\right),\;\;\Delta x_{\rho }=\Delta x_{E},\;\;x_{mid}=L_{2}.$$

### 4.4. Approximation of E(x) and E’(x) for ODE Model

The ODE “v” model requires gradient $E^{\prime }\left(x\right)$ for simulation to confirm the optimization result for each pin length *L*. To simplify E(x), and especially $E^{\prime }\left(x\right)$ for *Simulink* simulation, (20) will be approximated by $E_{p}\left(x\right)=E_{1}\sum_{i=0}^{10}p_{i}x^{i}$, fitted by the least-squares method.

## 5. Optimization of E(x) and ρ(x) Profiles

The model to be used in the *simplex* optimization procedure is defined in Section 3, followed by the optimization vector and its initial values in Section 4. The objective function and penalty functions are next to be defined. Since the goal is to get *f* and $x_{0}$ as close as possible to references $f_{ref}$ = 476 kHz and $x_{0ref}=L-d_{0}-d_{sch}$ for each pin length *L*, the main objective function is (26), with weight factors $k_{x}=10^{10}$ and $k_{f}=10^{-6}$.

(26) $$F_{obj}\;=\;k_{x}{\left(x_{0}-x_{0ref}\right)}^{2}\;+\;k_{f}{\left(f-f_{ref}\right)}^{2}\;$$

The first penalty function (27) is calculated from exponents $n_{E}$ and $n_{\rho }$. These exponents are calculated in each optimization step from values in the optimization vector *h*, according to (21). Penalty increases when $n_{E},n_{\rho }<2$ or when $n_{E},n_{\rho }>20$, as explained in Section 4.2. Factors $k_{E1}$ or $k_{\rho 1}$ have a value of 100 if $n_{E}$ < 2 or $n_{\rho }$ < 2, otherwise zero. Similarly, $k_{E2}$ or $k_{\rho 2}$ have a value of 10 if $n_{E}$ > 20 or $n_{\rho }$ > 20, otherwise zero.

$$F_{pn}=k_{E1}{\left(n_{E}-2\right)}^{2}+k_{E2}{\left(n_{E}-20\right)}^{2}+$$

(27) $$+k_{\rho 1}{\left(n_{\rho }-2\right)}^{2}+k_{\rho 2}{\left(n_{\rho }-20\right)}^{2}$$

The following penalty function (28) is introduced because the fast time integrator model (13) used in the optimization procedure loses precision if $x_{0}$ is located outside of the ($x_{1E}$,$x_{2E}$) or ($x_{1\rho }$,$x_{2\rho }$) range.

(28) $$F_{pm}\;=\;k_{m}{\left(\frac{x_{1E}+x_{2E}}{2}-x_{0}\right)}^{2}+k_{m}{\left(\frac{x_{1\rho }+x_{2\rho }}{2}-x_{0}\right)}^{2}\;$$

The optimization algorithm will hence be steered to move both arithmetic mean values towards $x_{0}$. The weight factor is set to $k_{m}=4\times 10^{8}$.

Boundary values $E_{1}$, $E_{2}$, $\rho_{1}$ and $\rho_{2}$ must stay within some reasonable range. This is why brass (the most common material for pins in pin-tumbler locks) was chosen as the initial point, and then the ultrasound speeds $v_{1}$ for the left-hand side of each pin were calculated, as described in Section 4 according to (19) and Figure 10. Although a much wider span of speeds of sounds is possible, ranging from 1200 m/s (common lead) to 20,000 m/s (diamond), the goal is to stay within a span of common, easily obtainable metal alloys, with a sufficient minimum shear strength (in the order of a common brass, i.e., 200 GPa).

**Figure 12:**
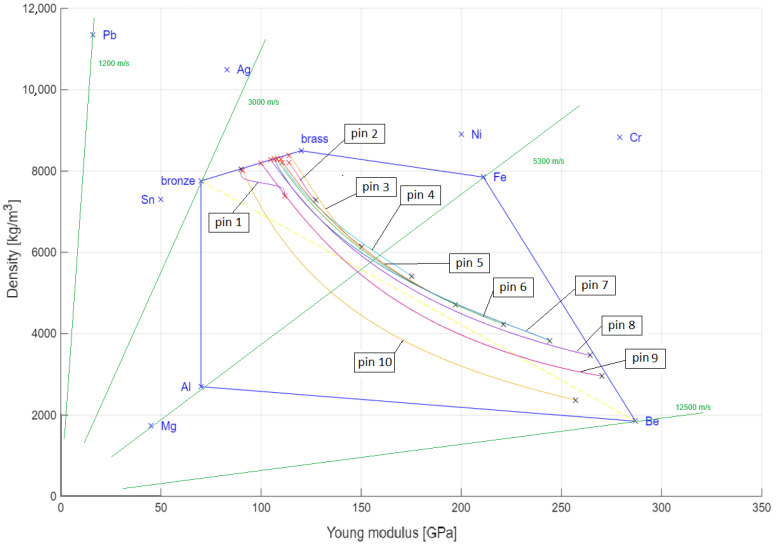
E-ρ diagram for optimized FGM key pins.

This is why a blue pentagon (Al-bronze-brass-Fe-Be) in Figure 12 is chosen as the permitted area. The following penalty function (29) will indicate if points $M_{1}$ = ($E_{1}$, $\rho_{1}$) or $M_{2}$ = ($E_{2}$, $\rho_{2}$) fall outside of the permitted area.

(29) $$F_{pE\rho }=k_{E}\rho \cdot \frac{\sum_{j=1}^{2}\sum_{i=1}^{5}P_{triangle,\;i\;}\left(E_{j},\rho_{j}\right)-{2P}_{pent}}{{2P}_{pent}}$$

The weight factor is $k_{E}\rho =2\times 10^{3}$. The constant $P_{pent}$ is the area of the pentagon. A sum of areas of five triangles is calculated for boundary points $M_{1}$ and $M_{2}$. For point $M_{1}$ its five triangles are ($M_{1}$-bronze-Al), ($M_{1}$-Al-Be), ($M_{1}$-Be-Fe), ($M_{1}$-Fe-brass), and ($M_{1}$-brass-bronze), and likewise for point $M_{2}$. If both boundary points are located within the pentagon, the sum of the areas of the five triangles equals the pentagon area, and the penalty function is zero. If a boundary point is located outside the pentagon, the sum of the areas of the five triangles is greater than the pentagon area, and the penalty function value (29) increases accordingly.

The cumulative penalty function is then calculated as a sum $F_{p}=F_{pn}+F_{pm}+F_{pE\rho }$.

According to [15,26], both Young’s modulus and density of a metal alloy (and a metal–ceramic composite as well) can be calculated (30) based on a simple rule-of-mixture with volume fractions $\phi_{i}$ as weight factors, assuming additivity of volume (Voigt model). The Voigt model is reasonably accurate for metal alloys and metal–ceramic composites, but not for porous materials [27], for which a more precise model (Mori-Tanaka [15]) must be used. This means any (*E*, ρ) combination within any triangle in Figure 12 can be achieved using a certain mixture of three or more metals.

(30) $$E\left(x\right)=\sum_{i=1}^{N}\phi_{i}\left(x\right)\cdot E_{i}\;\;\;\;,\;\;\;\rho \left(x\right)=\sum_{i=1}^{N}\phi_{i}\left(x\right)\cdot \rho_{i}$$

Boundaries of the pentagon in Figure 12 are set considering the following criteria:

(1) A sufficient shear strength for soft metals with *E* < 70 GPa is difficult to achieve, hence this is one of the limits. Duralumin with 95% aluminium will have a much higher shear strength than pure aluminium, but practically the same Young’s modulus and density, according to (30).
(2) The speed of sound in beryllium is very high (*v* = 12,500 m/s), and it is not expensive compared to the rest of the high-security lock, since it will be used to make only key pins. According to [28,29], alloys of beryllium, aluminium and iron are feasible in any ratio.
(3) The purpose of this paper is to prove that it is possible to calculate profiles of E(x) and ρ(x) for standard key pin lengths to achieve desired *f* and $x_{0}$, within a reasonable permitted range of E(x) and ρ(x), using reasonably priced and obtainable materials, considering the advances in production of various FGMs. Calculations of the exact composition of a pin’s alloys (to achieve a minimum shear strength and other important properties at every point) are beyond the scope of this paper.

### Results of Optimization Procedure Using Simplex Method

**Figure 13:**
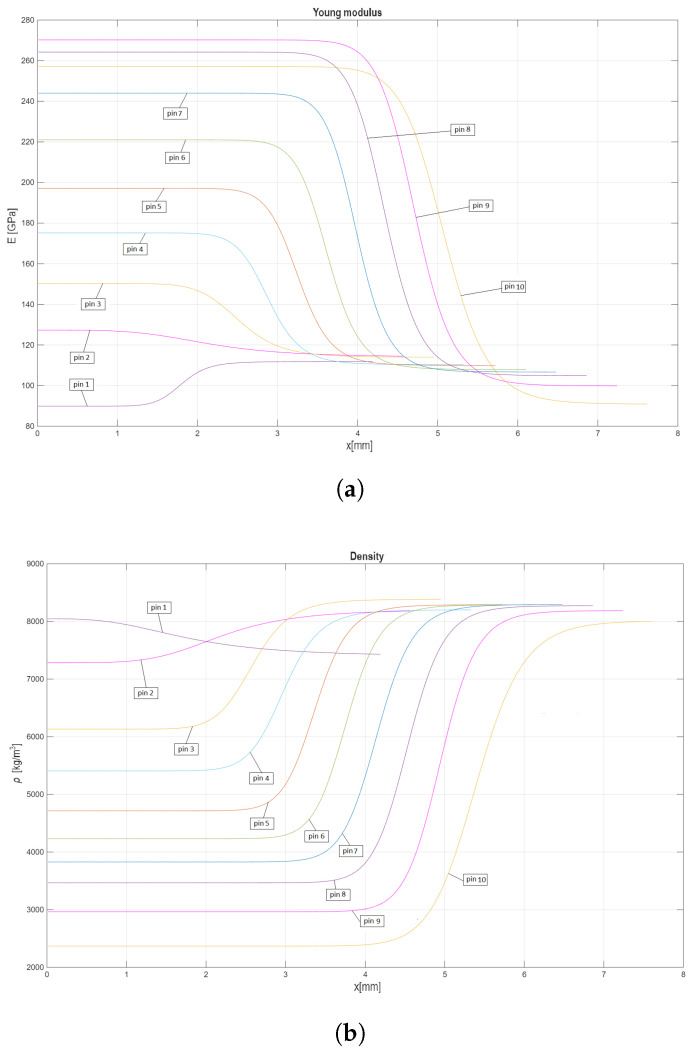
Optimized FGM profiles for 10 pin lengths: (**a**) E-x diagram, (**b**) ρ-x diagram.

The optimization procedure for all ten standard pin lengths, using the fast time integrator model (13), the objective function defined by (26), and penalty functions defined by (27)–(29), with initial values for each pin length set by (25), was performed. The resulting E(x) and ρ(x) profiles are shown in Figure 12 and Figure 13. The time needed for the optimization procedure in MATLAB, for all ten pins, is cca. 30 s.

The initial span of speeds of sound (19) calculated for two-section pins (Figure 10), as the initial values for *simplex* optimization, were 3410–9950 m/s. The speed span after the completed optimization (Figure 12) is 3350–10,450 m/s for pins with E(x) and ρ(x) modeled using Butterworth polynomials (20).

Red ‘X’ symbols on Figure 12 indicate right-hand, tapered pin tips, i.e., ($E_{2}$, $\rho_{2}$) points, with properties close to brass. Black ‘X’ symbols indicate left-hand ends or ($E_{1}$, $\rho_{1}$) points. FGM pin profiles between red ‘X’ and black ‘X’ are described by (20) and (21).

FEM simulation tests (Table 1 and Table 2) for all ten optimized pins indicate a maximum ±0.8% deviation in natural frequency from reference $f_{ref}$ = 476 kHz, and a maximum ±0.05 mm deviation in positions of standing wave nodes $x_{0}$ from references $x_{0ref}$.

## 6. Final Adjustments

The initial one-dimensional FEM, along with derived ODE and fast time integrator models, is valid for slender sticks, i.e., for L>2d. It can be used even in the range d<L<2d, but the natural frequency calculated from simulation results will thus be higher (because of short stick effects) than the actual natural frequency, according to [23,30]. Natural frequency for pin-1, pin-2 and pin-3 must therefore be corrected by increasing $E_{1}$ (and consequently $E_{2}$ and every value of E(x) calculated by *simplex* optimization) and decreasing $\rho_{1}$ (and therefore $\rho_{2}$ and ρ(x) profile) by a certain factor $k_{c}$.

(31) $$E_{c}\left(x\right)=E\left(x\right)\cdot k_{c}\;\;\;\;,\;\;\;\;\;\rho_{c}\left(x\right)=\rho \left(x\right)/k_{c}\;$$

The speed of ultrasound is thus increased by the $k_{c}$ factor, effectively increasing the actual pin’s natural frequency by the $k_{c}$ factor. The actual pins thus will all have the same natural frequency $f_{ref}$ = 476 kHz when short-stick effects are compensated. According to [23,30], the $k_{c}$ factor value for *Schlage* pin-1 is therefore $k_{c}$ = 1.023, for pin-2 is $k_{c}$ = 1.011 and for pin-3 is $k_{c}$ = 1.002. It can be neglected for all longer pins, from pin-4 to pin-10.

### 6.1. Introducing Random Fluctuations

Producing pins with exact E(x) and ρ(x) profiles as calculated by the *simplex* method (as in Figure 12) is an additional challenge, considering tolerances introduced by certain production methods. Some methods (e.g., metal powder 3D printing) may produce pins with slightly different natural frequencies *f*, which could still be correlated to their lengths.

This can be preventively remedied by additionally correcting every pin’s $E_{c}\left(x\right)$ and $\rho_{c}\left(x\right)$ profiles with a certain random factor $k_{rnd}$ calculated before production of each pin.

(32) $$E_{rnd}\left(x\right)=E_{c}\left(x\right)\cdot k_{rnd}\;\;\;\;,\;\;\;\;\;\rho_{rnd}\left(x\right)=\rho_{c}\left(x\right)/k_{rnd}\;$$

If a random number generator sets $k_{rnd}$ at 0.95 < $k_{rnd}$ < 1.05 range, thus introducing a random ±5% frequency fluctuation, this may be (depending on production tolerances) enough to fully obfuscate any possible remaining correlation between any pin’s length and its natural frequency. This is, in fact, an intentional increase in manufacturing tolerances.

### 6.2. Post-Processing After 3D Printing

According to chapter 5 of the book [15], DED 3D printers are capable of gradually mixing several metal powders at every layer. However, some imperfect steep layers along the *x*-axis after 3D printing can be expected to occur because of tolerances introduced by a certain type of 3D printer. Steep transitions always make ultrasonic attacks easier, as explained in Section 4.2. Alternatively, if the transitions between layers are too steep, they can be smoothed by applying a post-printing heat treatment. According to Fick’s laws of diffusion (also governing the processes of predeposition and redistribution of dopants in silicon in semiconductor production procedures), described in Section 5.1 of book [26], heating to a high temperature lower than the lowest melting point of alloyed metals (e.g., 700–900 °C) will cause a diffusion of metal atoms in both directions, effectively smoothing out the steep layer transitions.

## 7. Preliminary Experimentation

**Figure 14:**
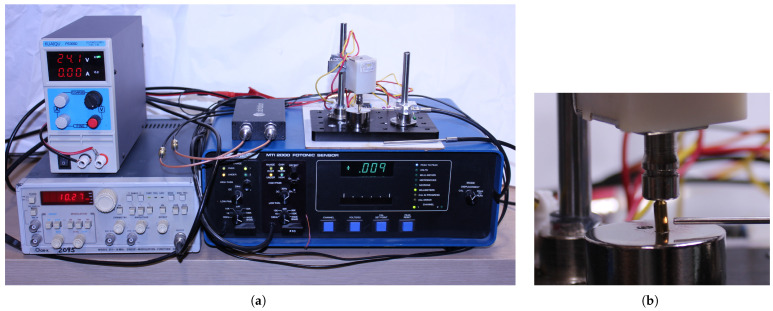
Experimental test setup: (**a**) whole rig, (**b**) brass pin under test illuminated by optic probe.

**Figure 15:**
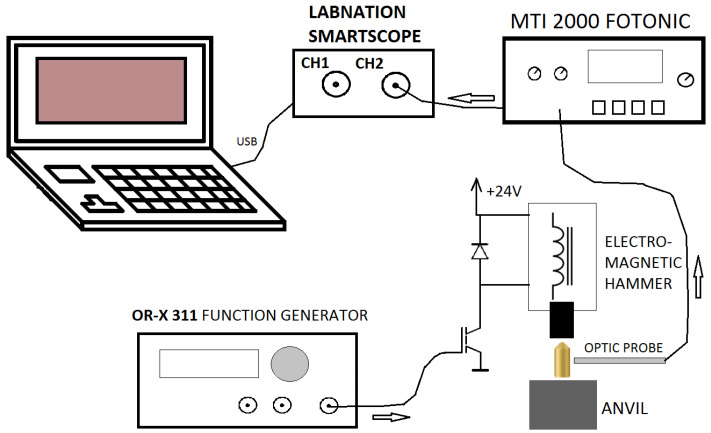
Schematic of experimental test setup.

The passive ultrasonic detector in Figure 1, outlined in [13], using a laser interferometry method (sensitive to phase shift, not only to amplitude of light), with a sufficient bandwidth (up to 1 MHz) is still under development, and will certainly give better experimental results than the currently available test rig in Figure 14 and Figure 15.

Since the MTI-2000 fiber-optic vibration/displacement detector uses a halogen incandescent bulb as a (incoherent) light source, it can register pin vibrations only by variations in amplitude, not in phase shift. Its upper corner frequency is 200 kHz, hence it can be expected to measure natural frequencies of brass pins longer than 8 mm, using a stronger excitation pulse.

An electromagnetic “hammer” can deliver a much stronger excitation than a pin spring inside a lock. It is controlled by squarewave pulses from the function generator. The brass pin under test has a length L = 9 mm. The chemical composition was analyzed using the XRF method at the KEEI chemical lab. It contains (mass fractions) 63% copper, 35% zinc and 2% lead. Assuming the additivity of volume, the volume fractions are 58.1%, 40.4%, and 1.5% respectively. Its Young’s modulus and density are then calculated from the Voigt model (30), and the speed of sound is hence *v* = 3710 m/s, which is lower than that of standard brass.

**Figure 16:**
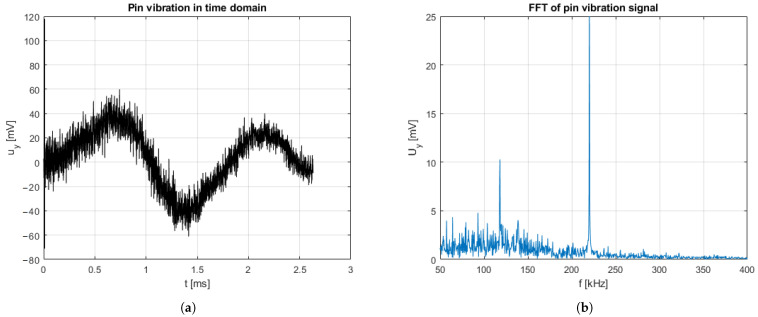
Response to the abrupt excitation at a point close to the tapered tip: (**a**) response in the time domain; (**b**) frequency spectrum of the signal.

**Figure 17:**
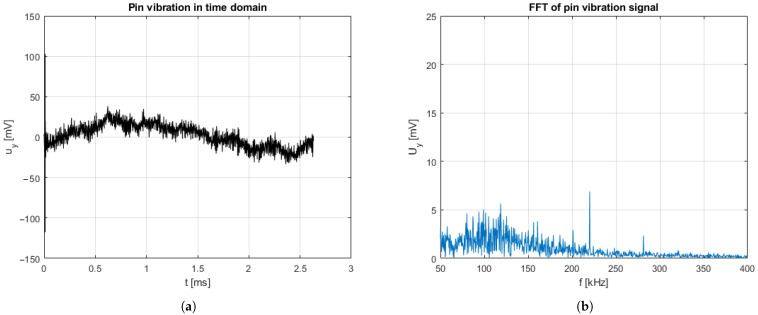
Response to the abrupt excitation at a point close to the standing wave node: (**a**) response in the time domain; (**b**) frequency spectrum of the signal.

The position of the optical probe can be adjusted by height, to a point with high amplitude (Figure 16, close to the tapered tip), or to the standing wave node (Figure 17). A natural frequency of the key pin with a tapered end was measured at *f* = 220 kHz as expected.

### Possible Experimental Problems and Challenges

FGM pins will be produced on a DED-type metal powder 3D printer. The MTI-2000 will be replaced in the test rig by a passive laser ultrasonic detector, for initial tests on brass and FGM pins, before attempting to measure their frequencies inside a pin-tumbler lock.

The initial assumption for the calculation of a pin’s natural frequency was that a pin behaves (most of the time after the abrupt excitation) as a mechanical transmission line with both ends free. In the worst case, if only one of its ends is tightly affixed, its natural frequency would be equal to half that calculated. This is a possible reason why signals with lower frequency will appear (like in Figure 16), because the termination impedances may vary (in less than a millisecond, both resistive and reactive) during impact. Higher harmonics may also be present.

To determine a pin’s length, the recorded signals must first be digitally processed. This requires a robust post-processing algorithm, ideally integrated with AI pattern recognition trained on diverse pin geometries. Such advanced processing is crucial because FGM pins of varying lengths can share the same natural frequency; however, they generate unique signal patterns that only sophisticated algorithms can distinguish.

Furthermore, resolving how to initiate a recording within a lock mechanism will be necessary. A probe scratching on the lock’s elements can generate abrupt signals. A signal recording will be initiated on a passive detector after each such signal, for about 5 ms, which is an average ring-out time. The recorded signal will have to be immediately checked on a passive detector’s MCU for the presence of periodic signals, and to distinguish between scratching and the actual pin ringout signal.

## 8. Conclusions

Mechanical locks are an important link in the chain of security. They are unlikely to ever be fully replaced by electric locks. Simple methods for quickly defeating fingerprint ID or RFID readers, or simply bypassing electric locks have already been discovered. Picking mechanical locks requires more training and manual dexterity. Mechanical locks are hence still being researched and improved, along with methods of attack.

Instead of using regular homogeneous metal alloys, it is proposed to manufacture a lock with key pins made of FGM alloys to make the lock more resistant to ultrasonic attacks. The key pins made of FGM alloys will thus have equal natural frequencies. Furthermore, the standing wave nodes on pins will be placed in the desired, most convenient positions.

The proposed method for calculating the profiles of FGM pins using the *simplex* optimization procedure with the fast time integrator model (to quickly calculate *f* and $x_{0}$) derived from the ODE “v” model needs less than one minute to complete calculations for 10 standard key pin lengths of any manufacturer. Although the original one-dimensional PDE and FEM models can have up to cca. +3% error for the shortest pins, it can be corrected by factors for certain L/d ratios. Additional randomness (for production techhniques like 3D printing) can easily be introduced to increase security.

Integrating FGM key pins provides heightened security against active and passive ultrasonic attacks at a higher cost (the price of FGM pins), while preserving the lock’s existing mechanical architecture.

Possible types of locks for further research are wafer locks (like Japanese *MIWA*) or disk-tumbler locks (like *DOM Diamant*). Unlike the pins in pin-tumbler locks, wafers and disks are two-dimensional elements. The next challenge for research is slider locks (for example, *EVVA 4KS*), since sliders are three-dimensional elements. Being 2D and 3D structures, mathematical models for wafers, disks and sliders will be significantly more complicated. Methods for precise production (like 3D printing) of pins, wafers, disks or sliders made of FGM alloys will also have to be further researched and improved in order to meet new demands.

## Figures and Tables

**Table 1:** Simulation tests—natural frequency, reference $f_{ref}$ = 476 kHz.

Frequency *f* [kHz]	Pin-1	Pin-2	Pin-3	Pin-4	Pin-5	Pin-6	Pin-7	Pin-8	Pin-9	Pin-10
FEM	475.50	475.50	475.00	475.00	475.00	474.50	474.00	474.00	473.50	472.50
ODE “v” model	475.52	476.01	476.06	476.21	476.21	476.35	476.45	476.60	476.40	475.77
Fast time-int model	476.30	476.29	476.29	476.29	476.29	476.29	476.29	476.29	476.29	476.29

**Table 2:** Simulation tests—standing wave node positions.

Node Position $x_{0}$ [mm]	Pin-1	Pin-2	Pin-3	Pin-4	Pin-5	Pin-6	Pin-7	Pin-8	Pin-9	Pin-10
Reference	1.79	2.17	2.55	2.93	3.32	3.70	4.08	4.46	4.84	5.22
FEM	1.802	2.175	2.565	2.942	3.329	3.709	4.095	4.459	4.865	5.270
ODE “v” model	1.793	2.175	2.564	2.932	3.318	3.697	4.069	4.431	4.836	5.258
Fast time-int model	1.785	2.166	2.554	2.931	3.318	3.697	4.069	4.445	4.836	5.227

## Data Availability

The original contributions presented in this study are included in the article. Further inquiries can be directed to the corresponding author.

## Abbreviations

The following abbreviations are used in this manuscript:

FEM	Finite-element model/method
FGM	Functionally graded materials
DED	Directed energy deposition
PDE	Partial differential equation
ODE	Ordinary differential equation
KEEI	Končar Electrical Engineering Institute
XRF	X-ray fluorescence
